# High-resolution deep learning-reconstructed T2-weighted imaging for the improvement of image quality and extraprostatic extension assessment in prostate MRI

**DOI:** 10.3389/fradi.2025.1695043

**Published:** 2025-10-31

**Authors:** Sebastian Gassenmaier, Franziska Katharina Staber, Stephan Ursprung, Judith Herrmann, Sebastian Werner, Andreas Lingg, Lisa C. Adams, Haidara Almansour, Konstantin Nikolaou, Saif Afat

**Affiliations:** ^1^ Department of Diagnostic and Interventional Radiology, Eberhard Karls University Tuebingen, Tuebingen, Germany; ^2^ Department of Diagnostic and Interventional Radiology, Klinikum Rechts der Isar, Technical University Munich, Munich, Germany; ^3^ Cluster of Excellence iFIT (EXC 2180) “Image-guided and Functionally Instructed Tumor Therapies”, University of Tuebingen, Tuebingen, Germany

**Keywords:** MRI, deep learning, prostate, mpMRI, high-resolution

## Abstract

**Purpose:**

This study evaluates the impact of high-resolution T2-weighted imaging (T2_HR_) combined with deep learning image reconstruction (DLR) on image quality, lesion delineation, and extraprostatic extension (EPE) assessment in prostate multiparametric MRI (mpMRI).

**Materials and methods:**

This retrospective study included 69 patients who underwent mpMRI of the prostate on a 3 T scanner with DLR between April 2023 and March 2024. Routine mpMRI protocols adhering to the Prostate Imaging Reporting and Data System (PI-RADS) v2.1 were used, including an additional T2_HR_ sequence [2 mm slice thickness, 4:31 min vs. 4:12 min for standard T2 (T2_S_)]. The image datasets were evaluated by two radiologists using a Likert scale ranging from 1 to 5, with 5 being the best for sharpness, lesion contours, motion artifacts, prostate border delineation, overall image quality, and diagnostic confidence. PI-RADS scoring and EPE suspicion were analyzed. The statistical methods used included the Wilcoxon signed-rank test and Cohen's kappa for inter-reader agreement.

**Results:**

T2_HR_ significantly improved lesion contours (medians of 5 vs. 4, *p* < 0.001), prostate border delineation (medians of 5 vs. 4, *p* < 0.001), and overall image quality (medians of 5 vs. 4, *p* < 0.001) compared to T2_S_. However, motion artifacts were significantly worse in T2_HR_. Substantial inter-reader agreement was observed in the PI-RADS scoring. EPE detection marginally increased with T2_HR_, though histopathological validation was limited.

**Conclusion:**

T2_HR_ imaging with DLR enhances image quality, lesion delineation, and diagnostic confidence without significantly prolonged acquisition time. It shows potential for improving EPE assessment in prostate cancer but requires further validation in larger studies.

## Introduction

Multiparametric magnetic resonance imaging (mpMRI) of the prostate is a key modality for the accurate detection and management of clinically significant prostate cancer (1). Given that prostate cancer is one of the most common solid cancers in men, the importance of mpMRI continues to grow (1–4). To ensure high diagnostic quality, the Prostate Imaging Reporting and Data System (PI-RADS) was established (5). The latest version, PI-RADS version 2.1, was published in 2019 (6). The guidelines recommend acquiring T2-weighted turbo spin-echo (T2w TSE) images with a slice thickness of at least 3 mm, together with key sequences such as diffusion-weighted imaging (DWI) and dynamic contrast-enhanced (DCE) imaging. This protocol provides sufficient insight into prostate pathologies.

However, detecting extraprostatic extension (EPE) can be challenging on MRI (7). Higher morphological resolution could potentially improve the diagnostic quality of prostate MRI (8, 9). The major drawback of higher resolution, however, is the increase in acquisition time (TA). mpMRI is already a lengthy examination due to the multiple sequences required. In addition, the predominantly elderly patient population undergoing prostate MRI often finds it hard to stay motionless for extended durations. The success of mpMRI in detecting prostate cancer has also led to an increasing demand for these examinations. This issue is further exacerbated by the aging population in Western countries, where a higher prevalence of prostate cancer is expected due to demographic changes.

To enhance the spatial resolution of T2-weighted imaging without further prolonging the TA, acceleration techniques are needed. These include conventional methods such as parallel imaging (PI) and compressed sensing (CS) (10, 11). However, the most powerful method currently available to compensate for the signal-to-noise ratio loss is deep learning image reconstruction (DLR) (12). Several studies have shown that DLR enables TA reduction without compromising image quality and, in some cases, even improving it (13, 14). This applies not only to genitourinary (GU) imaging but also to imaging of the chest, abdomen, and musculoskeletal system (13–19).

The aim of this study was to investigate the impact of high-resolution T2-weighted prostate imaging combined with DLR on image quality, lesion contours, assessment of EPE, and prostate border delineation, without a significant increase in TA.

## Materials and methods

### Study design

This monocentric, retrospective study was approved by the institutional review board with waiver of informed consent. All the study’s procedures were in line with the Declaration of Helsinki of 1964 and its later amendments.

All consecutive patients who underwent an mpMRI of the prostate due to suspicion of prostate cancer, staging of known prostate cancer, or active surveillance between April 2023 and March 2024 were included. The inclusion criteria were examination on a 3 T MRI scanner (Siemens MAGNETOM Vida^Fit^; Siemens Healthcare, Erlangen, Germany) with commercially available DLR software installed (Deep Resolve; Siemens Healthcare, Erlangen, Germany). Exclusion criteria were examination on an inappropriate scanner, incomplete imaging studies, and post-prostatectomy status.

### MRI acquisition parameters

The patients were examined in the supine position using a setup of an 18-channel body coil and 12 elements of a 32-channel spine coil. All the patients underwent our routine protocol, which adheres to the PI-RADS v. 2.1 and included the following sequences: standard T2w TSE imaging (T2_S_) in three planes (3 mm slice thickness), T1-weighted (T1w)-TSE axial imaging precontrast (3 mm slice thickness), DCE imaging [golden angle radial sparse parallel (GRASP) sequence, 3 mm slice thickness], DWI (b = 0 s/mm^2^, b = 1,000 s/mm^2^), and a T1w postcontrast radial gradient echo sequence (StarVIBE, 3 mm slice thickness). In addition, an axial T2w TSE sequence combined with DLR, with a slice thickness of 2 mm, was acquired (T2_HR_) in all cases after the standard T2 sequences. The TA of the standard 3-mm T2w imaging was 4:12 min as compared to 4:31 min for the 2-mm T2_HR_ combined with DLR. The detailed acquisition parameters of the T2w imaging are displayed in Table 1.

**Table 1:** MRI acquisition parameters of the T2_S_ and deep learning-reconstructed T2_HR_.

Parameter	T2_S_	T2_HR_
TR (ms)	8,930	5,950
TE (ms)	81	104
Concatenations	1	2
Average	3	2
Voxel size (mm)	0.5 × 0.5 × 3	0.3 × 0.3 × 2
Field of view (mm)	200	200
Slice thickness (mm)	3	2
Parallel imaging factor	3	4
Acquisition time (min:s)	4:12	4:31

T2_S_, standard T2-weighted imaging; T2_HR_, high-resolution T2-weighted imaging; TR, repetition time; TE, echo time.

### Deep learning image reconstruction technique

The principle of the applied DLR has been described in a previous study (14). In brief, we employed an unrolled variational network for MRI reconstruction that alternates trainable data-consistency steps with convolutional image-regularization blocks, conceptually extending compressed sensing by learning the regularizer from the data (20). The network ingests undersampled k-space data, coil sensitivity maps estimated from reference lines, and a normalization field for intensity homogenization, using conventional parallel imaging sampling patterns for acceleration. The reconstruction was explicitly designed to enhance the signal-to-noise ratio without altering the image contrast; consequently, the effects of the acquisition parameters (echo time, repetition time, and echo-train length) are identical to those of conventional reconstructions.

Supervised training was performed on approximately 10,000 turbo spin-echo slices acquired from volunteers on clinical 1.5 and 3 T systems (MAGNETOM, Siemens Healthcare, Erlangen, Germany) across multiple body regions (head, pelvis, and knee) and image contrasts. Training inputs were generated via retrospective fourfold undersampling with 75% phase resolution, and the loss combined an L1 term with a multiscale structural similarity (SSIM) component. The model was implemented in PyTorch, trained on a GPU system with 32 GB of memory, and then converted for deployment within a scanner-integrated inference framework. In routine use, the average per-slice inference time is approximately 3 s on the CPU and 0.5 s on the GPU.

### Image evaluation

All the imaging datasets (mpMRI with and without T2_HR_) were evaluated by two board-certified radiologists who both had a focus on GU imaging, with 6 and 10 years of experience, respectively. These evaluations were conducted independently, in a random order, and using a dedicated workstation (Centricity PACS RA 1000; GE Healthcare, IL, USA). Both readers were blinded to clinical data and histopathology. The datasets were evaluated in the following categories using a Likert scale ranging from 1 to 5, with 5 being the best: noise, sharpness, lesion contours, motion artifacts, prostate border delineation, overall image quality, and diagnostic confidence. The evaluation criteria were as follows: sharpness/lesion contours/prostate border delineation: 1–completely blurred, 2–hardly detectable imaging details, 3–slight blurring, 4–well-defined imaging details, and 5–excellent sharpness; noise: 1–very high level of noise, 2–elevated level of noise, 3–medium level of noise, 4–minimal level of noise, and 5–no noise; motion artifacts: 1–very pronounced motion artifacts, 2–pronounced motion artifacts, 3–intermediate motion artifacts, 4–minimal motion artifacts, and 5–no motion artifacts: overall image quality: 1–non-diagnostic, 2–severely hampered image quality, 3–intermediate quality, 4–good quality, and 5–excellent quality; diagnostic confidence: 1–non-diagnostic, repetition of examination recommended, 2–severely impaired image quality, repetition of examination recommended, 3–intermediate confidence, 4–good confidence, and 5–excellent confidence. Furthermore, PI-RADS scoring and suspicion of EPE were assessed. The sensitivity and specificity were calculated based on biopsy-confirmed cases. Clinically significant prostate cancer was defined as a Gleason score greater than 6. A PI-RADS score ≥3 was considered indicative of clinically significant cancer. Accordingly, lesions with a Gleason score of 6 and a PI-RADS score of 1 or 2 were classified as true negatives, whereas lesions with a Gleason score of 6 and a PI-RADS score of 3 were considered false positives. In cases with significantly different PI-RADS scoring between the readers (<3 vs. ≥3), a consensus reading was performed.

### Statistical analysis

Commercially available statistical software was used for the analyses (SPSS Statistics Version 29, IBM, Armonk, NY, USA). Parametric variables are displayed using mean ± standard deviation (SD). Non-parametric variables are displayed using median and interquartile range (IQR) in parentheses. The Wilcoxon signed-rank test for paired data was conducted for the ordinal-scaled variables and non-normally distributed variables. Cohen’s kappa was used to analyze the inter-reader agreement regarding the PI-RADS scoring and image quality. Furthermore, the intraclass coefficient (ICC) was calculated to analyze image quality. *P*-values below 0.05 were regarded as significant.

## Results

### Patients’ characteristics

**Figure 1:**
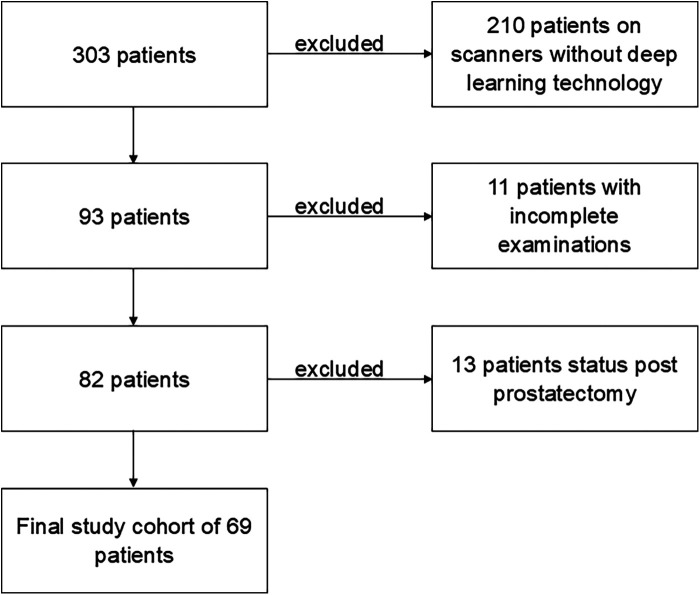
Flowchart of the study. In total, 303 patients were referred to an MRI scan of the prostate within 12 months, with 210 patients excluded due to examination on scanners without deep learning technology. Of the remaining 93 patients, 11 were excluded due to incomplete examinations. The final study cohort of 69 patients was the result of the exclusion of another 13 patients with post-prostatectomy status.

In total, 69 patients constituted the final study cohort (Figure 1). The mean patient age was 69 ± 7 years. In total, 51 patients underwent mpMRI due to suspicion of prostate cancer. Of these, 28 patients underwent a biopsy afterward, which revealed benign results in 10 cases. Finally, 11 patients underwent mpMRI for local staging of known prostatic cancer, and seven patients due to active surveillance. Further characteristics are shown in Table 2.

**Table 2:** Patients’ characteristics.

Characteristic	Value
Patient characteristics	
Number of patients	69
Mean age (years)	69 ± 7
Indication for MRI	
Suspicion of prostate cancer	51
Local staging of known prostate cancer	11
Active surveillance	7
Biopsy results	
Biopsies after MRI	29
Benign	10
Prostate cancer	19
Gleason grading	
Gleason 6	3
Gleason 7a	7
Gleason 7b	4
Gleason 8	3
Gleason 9	2
Prostatectomy results	
Prostatectomies	7
T-stage	
T2b	1
T2c	3
T3a	3

### Image quality

Inter-reader agreement was substantial between readers 1 and 2 (Cohen's kappa 0.632). The ICC between the readers was 0.706. For better readability, only the results of reader 1 are displayed in the following sections. All the results are shown in Table 3.

**Table 3:** Image quality in standard T2-weighted imaging (T2_S_) and deep learning-reconstructed high-resolution T2-weighted imaging (T2_HR_).

Characteristic	Reader 1			Reader 2		
	T2_S_	T2_HR_	*p*-value	T2_S_	T2_HR_	*p*-value
Sharpness	4 (4–4)	**5** (**4–5)**	<0.001	4 (4–5)	**4** (**4–5)**	0.001
Noise	5 (4–5)	**5** (**4–5)**	0.02	5 (4–5)	**5** (**5–5)**	<0.001
Lesion contours	4 (4–4)	**5** (**4–5)**	<0.001	4 (4–5)	**5** (**4–5)**	<0.001
Motion artifacts	**5** (**5–5)**	5 (4–5)	0.009	**5** (**5–5)**	4 (4–5)	<0.001
Prostate border delineation	4 (4–5)	**5** (**4–5)**	<0.001	4 (4–5)	**5** (**4–5)**	<0.001
Overall image quality	4 (4–5)	**5** (**4–5)**	<0.001	4 (4–5)	**5** (**4–5)**	<0.001
Diagnostic confidence	5 (4–5)	**5** (**5–5)**	0.005	5 (4–5)	**5** (**5–5)**	0.005

Statistically significant superior values printed in bold.

**Figure 2:**
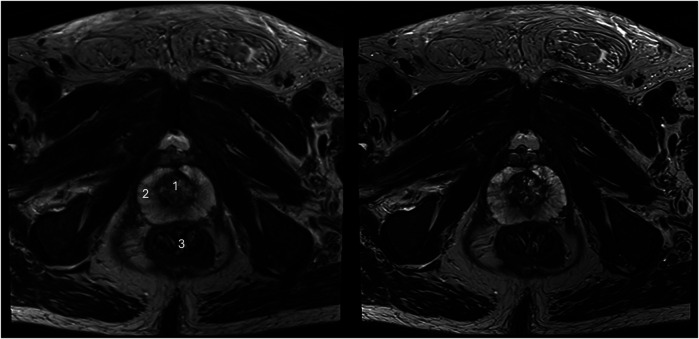
Imaging of a 73-year-old man with suspicion of prostate cancer. T2_S_ on the left-hand side and deep learning-reconstructed T2_HR_ on the right-hand side. T2_HR_ demonstrates a sharper depiction of the anatomical details and improved delineation of the transition and peripheral zones. The mpMRI was rated as PI-RADS 2 by both readers. (1) Transition zone of the prostate; (2) Peripheral zone of the prostate; (3) Rectum.

**Figure 3:**
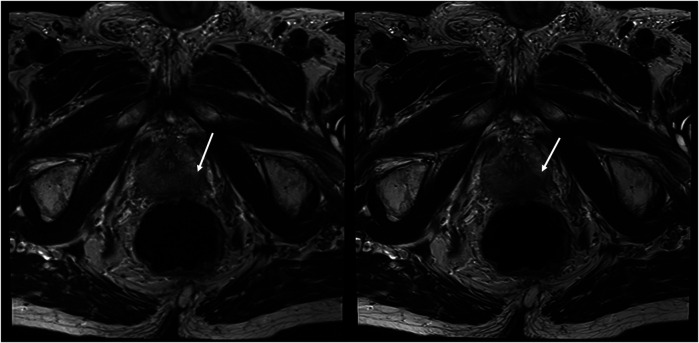
Imaging of a 78-year-old man with suspicion of prostate cancer. T2_S_ on the left-hand side and deep learning-reconstructed T2_HR_ on the right-hand side. T2_HR_ demonstrated improved delineation of the suspicious area (arrow). Both readers rated this case in both sequences as PI-RADS 5 with extraprostatic extension. The lesion size was 23 mm and the length of the capsule contact was approximately 16 mm.

**Figure 4:**
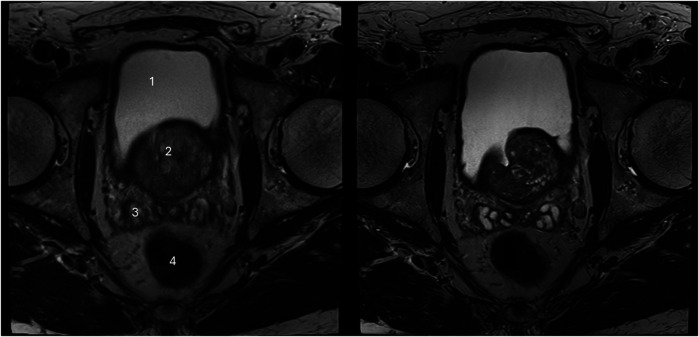
Imaging of a 71-year-old man with suspicion of prostate cancer. T2_S_ on the left-hand side and deep learning-reconstructed T2_HR_ on the right-hand side. The seminal vesicles are much sharper and have improved contrast in T2_HR_. No suspicious lesion was found by either reader (PI-RADS 2). (1) bladder; (2) transition zone of the prostate; (3) seminal vesicles; (4) rectum.

The sharpness of the prostate was evaluated to be significantly superior in T2_HR_ compared to T2_S,_ with medians of 5 (4–5) vs. 4 (4–4) (*p* < 0.001). The contours of the lesion were also rated superior, with a median of 5 (4 -5) in T2_HR_ vs. a median of 4 (4–4) (*p* < 0.001). The delineation of the prostate border was also rated to be improved in T2_HR_ [median of 5 (4–5)] as compared to T2_S_ [median of 4 (4–5); *p* < 0.001]. However, the extent of the motion artifacts was evaluated to be worse in T2_HR_ compared to T2_S_, with medians of 5 (4–5) vs. 5 (5–5) (*p* = 0.009). Please see Table 3 for the full details. Figures 2–4 show examples of both sequences.

### PI-RADS scoring and assessment of extraprostatic extension

The inter-reader agreement values for the PI-RADS scoring were 0.837 for T2_S_ and 0.857 for T2_HR_; thus, there was no significant intra-reader discrepancy in the PI-RADS scoring. Table 4 shows the details of the PI-RADS assessment. There was a significant discrepancy between the readers in two cases (PI-RADS 2 vs. PI-RADS 3). After consensus readings, both cases were assessed to have a PI-RADS 2. In total, 29 patients underwent a biopsy after the MRI scan, with benign results in 10 cases. In three of these benign cases, a PI-RADS score of 4 was given by both readers and, in one case, a PI-RADS score of 3 was given by both. Moreover, 19 biopsies revealed prostatic cancers, of which 17 cases had been previously suspected by both readers with PI-RADS scores of 4 or 5. One case of carcinoma was assessed to have a PI-RADS score of 2 by both readers (after consensus reading; Gleason score of 6). Furthermore, the sensitivity and specificity of the 29 biopsy cases were evaluated. Sensitivity and specificity were 100% and 46%, respectively, for T2_S_ and T2_HR_. Extraprostatic extension was suspected by reader 1 in 14 cases in T2_S_ and in 16 cases in T2_HR_, and reader 2 suspected EPE in 13 cases in T2_S_ and in 15 cases in T2_HR_. Histopathological correlation was only available in eight cases, with a prostatectomy revealing EPE in three cases. In one of these cases, EPE was suspected by both readers in T2_S_ and T2_HR_. In the second case, EPE was only suspected in T2_HR_ by both readers, and in the third case, no suspicion of EPE was raised by either reader.

**Table 4:** PI-RADS scoring.

Score	Reader 1		Reader 2	
	T2_S_	T2_HR_	T2_S_	T2_HR_
PI-RADS 2	24	24	26	26
PI-RADS 3	8	8	6	6
PI-RADS 4	21	22	21	21
PI-RADS 5	16	15	16	16

## Discussion

This study investigated the impact of T2_HR_ prostate imaging combined with DLR on image quality, lesion contours, prostate border delineation, and EPE assessment. Our findings indicate that T2_HR_ improves overall image quality and may enhance the detection of EPE, a critical factor in determining appropriate therapeutic strategies for prostate cancer.

The assessment of EPE is of utmost importance for patient management and therapeutic decisions (21, 22). In particular, the detection of EPE of transition zone cancer is very challenging, with higher missed detection rates compared to peripheral zone cancers (21). The presence of EPE is an established predictor of worse outcomes in prostate cancer patients (23). However, the sensitivity of mpMRI is limited regarding an exact assessment of EPE (7). Our study suggests that T2_HR_ imaging, by improving prostate border delineation, could offer a potential solution to these limitations, enhancing the sensitivity of EPE detection. However, this hypothesis requires validation in larger studies, as only eight patients in our cohort underwent a prostatectomy, limiting our ability to confirm the findings through histopathological correlation. Future studies should address this gap by including a greater number of prostatectomy cases, with MRI performed as close to surgery as possible to ensure an accurate comparison between the imaging and surgical results. A possible study concept would be to perform an mpMRI in all patients who are scheduled for a prostatectomy. Ideally, all prostatectomy specimens should undergo a complete histopathological evaluation.

Beyond its potential for improving EPE detection, T2_HR_ demonstrated clear benefits in overall image quality, lesion clarity, and radiologist confidence. High diagnostic confidence is essential not only for detecting clinically significant cancers but also for minimizing the overdiagnosis of non-significant cancers. This could lead to more precise patient management, reducing unnecessary treatments and their associated side effects. Beyond higher reader confidence, T2_HR_ improved lesion contours and prostate border delineation, which can aid biopsy planning (more precise target definition) and local staging/surgical strategy. However, the slightly longer TA of T2_HR_ led to increased motion artifacts, particularly because the sequence was performed after standard T2 and diffusion-weighted imaging. Apart from the sequence order, the higher extent of motion artifacts could also be due to the lower number of signal averages.

This study also underscores the evolving role of individualized, patient-centered imaging approaches in radiology. With DLR, it is possible to achieve high-resolution imaging without significantly increasing the TA, making it a valuable tool for patients who can remain still for extended periods. For patients with limited compliance, shorter sequences with standard resolution combined with DLR could be a better option to maintain diagnostic quality while minimizing motion artifacts. Furthermore, using these ultra-fast sequences, repeat scans due to insufficient image quality may be avoided, leading to improved workflow. This flexibility in imaging protocols supports the broader goal of personalized medicine, ensuring that each patient receives the most appropriate diagnostic strategy based on their unique needs.

Our findings are in line with existing studies on the benefits of DLR across a range of MRI applications, including musculoskeletal, abdominal, and thoracic imaging, which mainly focused on TA reduction (19, 24–29). This article also supports previous studies that demonstrated the advantages of DLR in prostate imaging (29–31). In comparison to previous studies, this study also evaluated, apart from image quality metrics, the presence of EPE, using histopathology as the reference standard (14, 15). The ability of DLR to substantially reduce TA without sacrificing image quality—in some cases, even enhancing it—is a significant advancement. While PI and CS have long been used to accelerate MRI, the extent of time reduction without loss of image quality achieved with DLR is unmatched (12). This makes DLR a crucial innovation in addressing the growing demand for MRI, particularly in settings where scanner availability is limited and patient volumes are increasing.

The main limitation of this study is the small number of patients who underwent a prostatectomy, strongly restricting the ability to fully assess EPE using histopathology. A statistically significant difference may be revealed when using larger sample sizes. Therefore, larger studies are necessary to further investigate this matter. In addition, DLR was only applied to T2_HR_ imaging in axial orientation due to TA considerations. Furthermore, DLR for DWI was not available for our scanner during the study period. Future studies should explore the benefits of incorporating DLR into other sequences, such as standard-resolution T2 and DWI, to further enhance overall image quality and diagnostic accuracy across the entire mpMRI protocol. Another limitation is the large number of patients who were excluded from this study due to examination on an inappropriate scanner, which may represent a selection bias. As only one scanner was included in this study, the findings demonstrated limited generalizability to other scanners and vendors. Furthermore, the study cohort was heterogeneous due to the inclusion of those with different indications for MRI, namely, staging, active surveillance, and suspicion of cancer. Sensitivity and specificity were only calculated on a small subset, with histopathology as the reference standard.

In conclusion, our study demonstrates the positive effect of high-resolution T2-weighted imaging with DLR on image quality, lesion contours, and diagnostic confidence. The potential for enhancing EPE detection using T2_HR_ combined with DLR is promising; however, further investigations with larger patient cohorts in future studies to confirm this hypothesis are warranted. The adjustment of the scanning protocol using high-resolution and standard-resolution sequences based on the patient's need represents a significant advancement in personalized medicine in radiology.

## Data Availability

The original contributions presented in the study are included in the article/Supplementary Material, further inquiries can be directed to the corresponding author.
